# Predicting amyloid status in corticobasal syndrome using modified clinical criteria, magnetic resonance imaging and fluorodeoxyglucose positron emission tomography

**DOI:** 10.1186/s13195-014-0093-y

**Published:** 2015-03-02

**Authors:** Sharon J Sha, Pia M Ghosh, Suzee E Lee, Chiara Corbetta-Rastelli, Willian J Jagust, John Kornak, Katherine P Rankin, Lea T Grinberg, Harry V Vinters, Mario F Mendez, Dennis W Dickson, William W Seeley, Marilu Gorno-Tempini, Joel Kramer, Bruce L Miller, Adam L Boxer, Gil D Rabinovici

**Affiliations:** Department of Neurology and Neurological Sciences, Stanford University, 300 Pasteur Drive, Rm A343, Stanford, CA 94305 USA; Department of Neurology, University of California, San Francisco, San Francisco, CA USA; Helen Wills Neuroscience Institute, University of California, Berkeley, Berkeley, CA USA; Lawrence Berkeley National Laboratory, Berkeley, CA USA; Department of Epidemiology and Biostatistics, University of California, San Francisco, San Francisco, CA USA; Department of Laboratory Medicine & Pathology, Mayo Clinic, Jacksonville, FL USA; Department of Neurology, University of California, Los Angeles, CA USA; Department of Pathology and Laboratory Medicine, University of California, Los Angeles, CA USA

## Abstract

**Introduction:**

Group comparisons demonstrate greater visuospatial and memory deficits and temporoparietal-predominant degeneration on neuroimaging in patients with corticobasal syndrome (CBS) found to have Alzheimer’s disease (AD) pathology versus those with underlying frontotemporal lobar degeneration (FTLD). The value of these features in predicting underlying AD pathology in individual patients is unknown. The goal of this study is to evaluate the utility of modified clinical criteria and visual interpretations of magnetic resonance imaging (MRI) and fluorodeoxyglucose positron emission tomography (FDG-PET) for predicting amyloid deposition (as a surrogate of Alzheimer’s disease neuropathology) in patients presenting with CBS.

**Methods:**

In total, 25 patients meeting CBS core criteria underwent amyloid (Pittsburgh compound B; PIB) PET scans. Clinical records, MRI, and FDG scans were reviewed blinded to PIB results. Modified clinical criteria were used to classify CBS patients as temporoparietal variant CBS (tpvCBS) or frontal variant CBS (fvCBS). MRI and FDG-PET were classified based on the predominant atrophy/hypometabolism pattern (frontal or temporoparietal).

**Results:**

A total of 9 out of 13 patients classified as tpvCBS were PIB+, compared to 2out of 12 patients classified as fvCBS (*P* < 0.01, sensitivity 82%, specificity 71% for PIB+ status). Visual MRI reads had 73% sensitivity and 46% specificity for PIB+ status with moderate intra-rater reliability (Cohen’s kappa = 0.42). Visual FDG reads had higher sensitivity (91%) for PIB+ status with perfect intra-rater reliability (kappa = 1.00), though specificity was low (50%). PIB results were confirmed in all 8 patients with available histopathology (3 PIB+ with confirmed AD, 5 PIB- with FTLD).

**Conclusions:**

Splitting CBS patients into frontal or temporoparietal clinical variants can help predict the likelihood of underlying AD, but criteria require further refinement. Temporoparietal-predominant neuroimaging patterns are sensitive but not specific for AD.

**Electronic supplementary material:**

The online version of this article (doi:10.1186/s13195-014-0093-y) contains supplementary material, which is available to authorized users.

## Introduction

Corticobasal syndrome (CBS) is a neurodegenerative syndrome characterized by asymmetric rigidity, apraxia, dystonia, loss of voluntary limb control and cortical sensory loss [1-3]. Although CBS was once thought to reliably predict neuropathological findings of corticobasal degeneration (CBD), autopsy studies have revealed diverse pathological substrates for CBS, including progressive supranuclear palsy, Lewy body disease (LBD), frontotemporal lobar degeneration with TDP-43 inclusions (FTLD-TDP) and prion disease. Alzheimer’s disease (AD) is found to be the primary underlying pathology in approximately 25% of patients [4-8]. Identifying patients with underlying AD during life is important because they may benefit from symptomatic therapies and future disease modifying agents. Patients with primary or co-morbid AD may also be excluded from clinical trials of biologically specific therapies in CBS in order to avoid potential confounds of amyloid co-pathology.

As a group, patients with CBS due to AD (CBS-AD) show greater episodic memory loss and visuospatial deficits, whereas CBS patients due to FTLD pathology (CBS-FTLD) show more behavioral changes, executive dysfunction, non-fluent aphasia and orobuccal apraxia [7,9-11], although these differences are not always found [12]. On neuroimaging, CBS-AD patients demonstrate greater temporoparietal involvement, while CBS-FTLD patients display greater brainstem atrophy [7,10,11]. Both groups show atrophy in a common peri-rolandic network thought to underlie the core CBS syndrome [7,10,11]. Similar group-level differences in clinical features and atrophy patterns were recently reported using amyloid imaging as a surrogate for AD pathology [13]. The value of these observations for predicting pathology in individual patients has not been investigated.

In this study, we evaluated whether splitting CBS into frontal (fvCBS) and temporoparietal-predominant variants (tpvCBS) based on clinical features could distinguish patients with FTLD versus AD pathology. Although an expert international group recently proposed new diagnostic criteria for CBD [14], the new criteria were published after completion of the chart review phase of our study such that we were not able to test their reliability prospectively and in blinded fashion. Because the majority of our autopsied patients were included in a previous report that demonstrated group-level differences between CBS-AD and CBS-FTLD [7], we tested the predictive value of CBS variants in an independent cohort of patients who underwent positron emission tomography (PET) imaging with the beta-amyloid ligand Pittsburgh compound B (PIB-PET) as a surrogate marker of AD pathology [15]. Eight patients included in the study have subsequently undergone pathologic examination. Although amyloid PET scans may ultimately be the optimal imaging modality for detecting AD pathology in CBS patients, this technology is not yet widely available or reimbursed by third-party payers. We, therefore, sought to determine whether more accessible neuroimaging modalities, such as structural magnetic resonance imaging (MRI) and fluorodeoxyglucose PET (FDG-PET), could, individually, predict PIB-PET status. Finally, we investigated whether combinations of clinical and imaging features could further improve our ability to distinguish CBS-AD and CBS-FTLD during life.

## Methods

### Standard protocol approvals, registrations, and patient consents

All aspects of the study were approved by the institutional review boards at the University of California San Francisco (UCSF), UC Berkeley, Lawrence Berkeley National Laboratory (LBNL), UC Los Angeles, and Mayo Clinic Jacksonville. All patients or surrogates provided written informed consent.

### Participants

#### Patients

A search in the UCSF Memory and Aging Center database identified 31 patients with a clinical diagnosis of CBS who underwent PIB-PET between 2005 and 2013. One patient was excluded due to an incomplete PIB-PET scan and four were excluded because chart review (by SJS, see below) revealed that they did not meet core CBS research criteria (Table 1). One patient was excluded because the rater knew the patient and could not be blinded. All patients included in the study underwent a history and examination by a neurologist with expertise in neurodegenerative disease, cognitive testing by a neuropsychologist and a structural MRI and/or FDG-PET.

**Table 1:** **Modified corticobasal syndrome criteria**

**I. CBS: core criteria**		
Inclusion criteria (1 plus 2)		
	1. Progressive course	
	2. At least three of the following:	
		a. Parkinsonism (bradykinesia or rigidity)
		b. Dystonia
		c. Myoclonus
		d. Impairment in voluntary limb control (alien limb)
		e. Cortical sensory deficit (symmetric/asymmetric)
Exclusion criteria (all must be negative)		
		a. Visual hallucinations
		b. REM sleep behavior disorder
		c. Cerebellar ataxia
		d. Prominent autonomic dysfunction
		e. Fluctuations in alertness
		f. Prominent rest tremor
**II. CBS- frontal variant:**		
Inclusion criteria (meets core plus one of the following must be positive)		
	1. Agrammatism, non-fluent speech or motor speech deficits	
	2. Apathy, disinhibition or loss of empathy	
	3. Apraxia primarily affecting lower extremities	
	4. Prominent executive dysfunction greater than memory or visuospatial impairment	
**III. CBS- temporoparietal variant:**		
Inclusion criteria (meets core plus one of the following must be positive)		
	1. Logopenic aphasia [16]	
	2. Elements of Gerstmann or Balint syndrome	
	3. Episodic memory or visuospatial impairment greater than executive dysfunction	

#### Controls

Age-matched cognitively-normal controls (NC) recruited from the community who had undergone both FDG-PET and PIB-PET as part of aging research studies were used as imaging controls [17]. To try to best match our patient group, we selected a comparably sized group of the youngest available controls with FDG data. Controls were not selected based on PIB results – however, 3/26 were PIB+ applying a global quantitative threshold of distribution volume ratios (DVR) ≥1.20 [18]. On retrospective assessment of FDG scans, none of these individuals showed an AD-like pattern or other qualitatively apparent abnormalities.

A total of 25 CBS patients and 26 normal controls were included in further analyses.

### Procedures

#### Core and variant criteria

Core CBS clinical criteria were modified from our previously published report [19]. We defined specific clinical features associated with fvCBS or tpvCBS (Table 1). The criteria were based on our previous group-level analysis [7], review of the literature, and were agreed on by clinicians experienced in CBS evaluation (SJS, SEL, ALB, KR, JHK, WWS, MGT, BLM, GDR).

#### Blinded chart review

A neurologist (SJS) who was blinded to imaging results reviewed clinical summaries and cognitive test results from each patient’s UCSF evaluation. Each diagnostic criterion was noted as present or absent for the patient’s initial visit. Based on the criteria (Table 1), patients were classified as fvCBS or tpvCBS. If a patient met criteria for both variants, the rater used her overall impression to assign one variant. All diagnoses were given a confidence rating using a 5-point scale: 1-highest confidence of fvCBS, 2-moderate confidence of fvCBS, 3-equivocal rating, 4-moderate confidence tpvCBS, 5-highest confidence of tpvCBS.

#### Genetics and neuropathology

APOE genotyping was performed on patients and controls (Table 2). Five patients had autopsies performed using previously published protocols [7] and one had a biopsy at UCSF. One autopsy was performed at Mayo Clinic Jacksonville and one was performed at UCLA using previously published methods [10,20]. Consensus criteria were used for the pathological diagnosis of AD [21], CBD [22] and LBD [23].

**Table 2:** **Demographics**

**Syndromic diagnosis**	**CBS-PIB + (number = 11)**	**CBS-PIB - (number = 14)**	**NC (number = 26)**	***P***	***Post hoc***
Age at PET	66.9 ± 5.5	65.8 ± 8.8	70.2 ± 2.9	0.054	
Age at MRI	67.2 ± 5.2	66.1 ± 9.3 (n = 13)		0.110	
Age at first clinic visit	65.6 ± 9.1	66.5 ± 5.1	n/a	0.750	
Male: Female	6:5	6:8	14:12	0.810	
Education, years	15.7 ± 2.2	15.8 ± 2.4	17.3 ± 2.0	0.620	
MMSE	22.9 ± 6.3	22.4 ± 6.9	29.2 ± 1.2	0.0001	CBS-PIB+ vs NC *P* <0.05,
					CBS-PIB- vs NC *P* <0.01
CDR	0.7 ± 0.5	0.9 ± 0.6	n/a	0.830	
CDR-SB	4.7 ± 3.2	3.7 ± 2.6	n/a	0.400	
*APOE4* (0,1,2)	4, 6, 0 (n = 10)	9, 1, 0 (n = 10)	17, 8, 1	0.066	CBS-PIB+ vs CBS-PIB- *P* < 0.05

CBS-PIB+, Corticobasal syndrome positive for Pittsburgh compound B; CBS-PIB-, Corticobasal syndrome negative for Pittsburgh compound B; CDR, Clinical Dementia Rating; CDR-SB, Clinical Dementia Rating sum of boxes; MMSE, Mini-mental State Examination; n/a, not applicable; NC, normal control. Data are mean ± standard deviation.

#### Image acquisition

Patients underwent high-resolution (1 mm^3^) T1-weighted structural MR imaging at UCSF (3 T scanner) or at the San Francisco Veterans Affairs Medical Center (1.5 T or 4 T scanner), as previously described [24-26]. MRI scans were acquired within one year of PET scan. PET imaging with [^11^C]PiB and [^18^F]FDG were performed on a Siemens ECAT EXACT HR (N = 22) or Siemens Biograph PET/CT Truepoint 6 (N = 1) scanner at LBNL. Image preprocessing and analysis were performed using Statistical Parametric Mapping version 8 [27]. FDG-PET frames for each subject were summed and standard uptake volume ratios (SUVR) were normalized to mean activity in the pons. PIB-PET DVR were calculated with Logan graphical analysis [28] using the grey matter cerebellum time-activity curve as reference tissue input function [29]. Mean FDG SUVR from ‘frontal,’ ‘temporoparietal’ and ‘common’ (the latter reflecting peri-rolandic regions affected across CBS variants) cortical regions of interest (ROIs) were extracted in template space using the Automated Anatomical Labeling (AAL) atlas space as previously described [30]. The ROIs (separately for left and right hemispheres and combined) were created using the AAL Atlas [31] and included: frontal cortex (composed of AAL regions superior frontal gyrus, middle frontal gyrus, inferior frontal gyrus, operculum, olfactory gyrus, gyrus rectus, insula, anterior cingulate), temporoparietal cortex (AAL regions posterior cingulate, superior parietal lobe, inferior parietal lobe, supramarginal gyrus, angular gyrus, precuneus, superior temporal gyrus, middle temporal gyrus, inferior temporal gyrus) and CBS common regions (AAL regions precentral gyrus, postcentral gyrus, supplementary motor area, paracentral lobule).

#### Image visual rating

**Figure 1:**
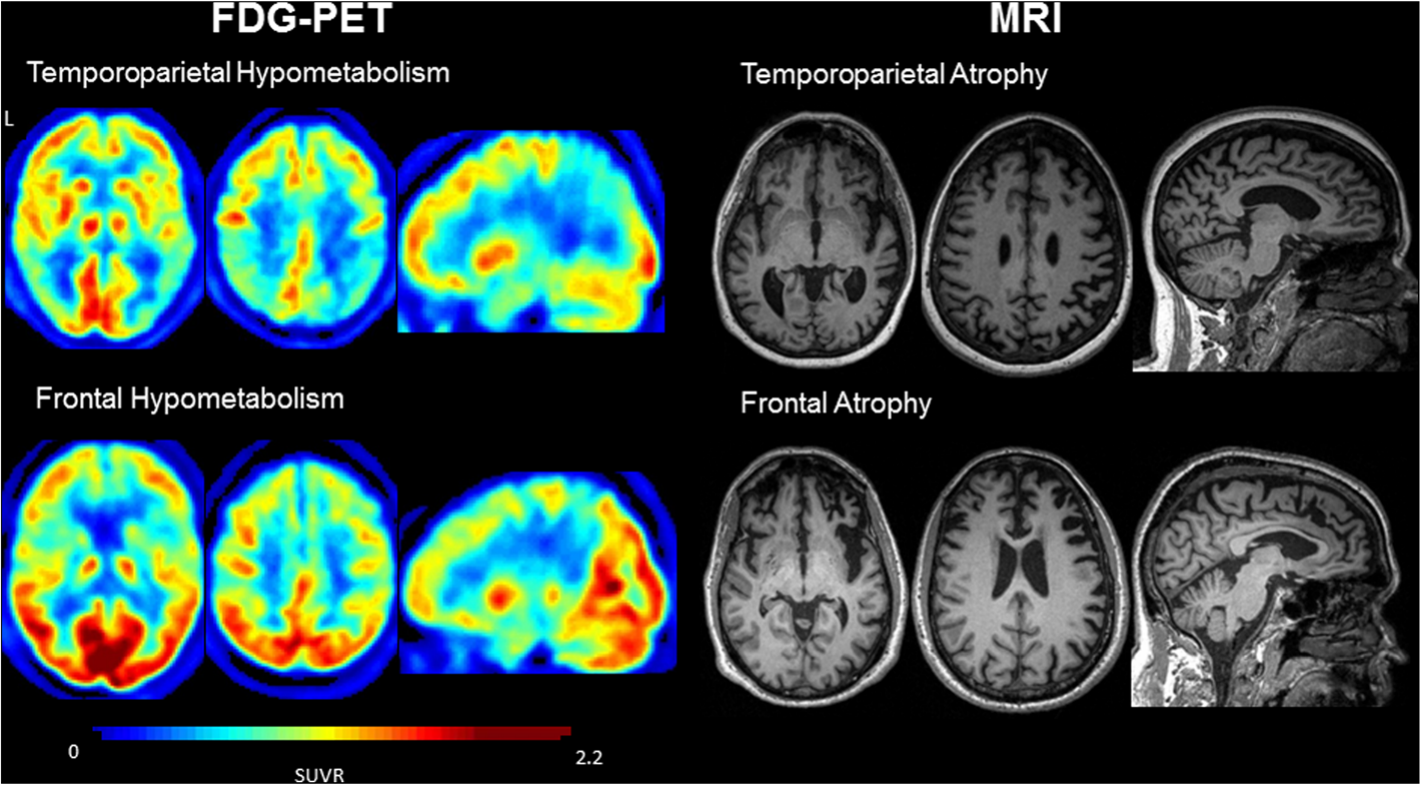
**Typical FDG PET metabolic patterns and MRI atrophy patterns.** FDG, fluorodeoxyglucose; MRI, magnetic resonance imaging; PET, positron emission tomography.

A neurologist (SJS) visually rated MRI scans as either ‘frontal variant’ (fv) or ‘temporoparietal variant’ (tpv) corresponding to an atrophy pattern greatest in frontal regions or temporoparietal regions, respectively (see Figure 1 for typical atrophy patterns). Each scan was given a confidence rating using the same scale described for clinical diagnosis. [^18^F]FDG PET scans were visually rated at a separate session in an analogous manner (SJS) blinded to diagnosis and PIB-PET status (Figure 1 for typical hypometabolic patterns). Intra-rater reliability was ascertained by re-reading randomly determined subsets of 10 MRI and 10 FDG-PET scans at a separate sitting, blinded to prior rating.

#### FDG-PET quantitative rating

Quantitative ratings of FDG-PET were performed to compare the qualitative interpretations performed in routine clinical practice with more rigorous quantitative approaches to see if the latter provide added value. FDG uptake in each ROI was assigned a Z score based on regional uptake in the normal control group. Classification was determined by the lowest ROI Z score for each patient (for example, tpv was classified if Z score was lower in temporoparietal regions than in frontal region). The difference in Z scores was calculated as the Z difference = Z (frontal) – Z (temporoparietal) [18]. To assess for asymmetry, lateralization indices (LIs) were calculated to compare left- and right-sided tracer uptake in ROIs according to the following formula: abs((right side/left side))-1 × 100. Quantitative assessments of MRI data were not done because patients were imaged at different magnet strengths.

#### Pittsburgh compound B status

PIB scans from CBS patients were visually rated as positive or negative for cortical uptake (by WJJ or GDR) blinded to all other clinical data [18]. Each patient was classified as CBS-PIB positive (CBS-PIB+) or CBS-PIB negative (CBS-PIB-). Ratings of clinical criteria, FDG-PET and structural MRI were compared to the PIB-PET status as the gold standard for the presence or absence of significant amyloid burden.

#### Voxel-wise group comparisons

To reproduce group-level differences in the anatomic pattern of neurodegeneration previously observed in autopsy-proven patients of AD and FTLD pathology [7], we compared FDG patterns in CBS-PIB+ and CBS-PIB- compared to NC and to each other. Voxel-wise comparisons of FDG SUVR images were performed in SPM8 using an analysis of covariance model that included diagnosis (controls, CBS-PIB+, CBS-PIB-) as the condition, and age, sex and education as covariates. Pairwise contrasts were performed among the three groups as follows: CBS-PIB+ < NC, CBS-PIB- < NC, CBS-PIB+ > CBS-PIB-, and CBS-PIB- > CBS-PIB+. Resulting T-maps were displayed on an MNI template brain at a threshold of *P* <0.001, uncorrected for multiple comparisons.

### Statistical analysis

Group differences in dichotomous variables were compared using Chi-square or Fisher’s exact test. Group differences in continuous variables were evaluated using analysis of variance with *post hoc* Gabriel’s procedure or Games-Howell procedure, depending on variance of data. For non-parametric data, Mann–Whitney tests were applied for comparisons between two groups and Kruskal-Wallis tests for three groups. Intra-rater reliability was measured using Cohen kappa statistic. Sensitivity, specificity, and positive and negative predictive values were estimated by the appropriate observed proportion and 95% confidence intervals were generated based on the assumption that they follow a binomial distribution. Odds ratio (OR) was employed to determine increased risk of PIB positivity for clinical criteria. Statistical analysis was implemented in Predictive Analytics SoftWare 20.0 (SPSS Inc.).

## Results

### Demographics

Patients and controls were matched for education and sex (Table 2). There was a trend for older age at the time of PET in the control group due to the older targeted age recruitment for controls at our center. Patients were more impaired than controls (Mini Mental State Exam (MMSE)), but there were no differences between CBS-PIB+ and CBS-PIB- (MMSE, Clinical Dementia rating). Six of ten CBS-PIB+ but only one of ten CBS-PIB- patients carried the apolipoprotein E ε4 allele (P = 0.029).

### CBS criteria

All patients met CBS criteria for either fv or tpv. Nineteen patients met fvCBS criteria, eighteen met tpvCBS criteria and twelve met criteria for both, requiring a designation by the clinician (see Additional file 1: Table S1). Thirteen patients had a final designation of tpv and twelve had fv. Five of 12 and 5/13 were given highly confident ratings for fvCBS and tpvCBS, respectively. There were no differences in confidence ratings between the two groups.

Eleven patients were PIB+ and fourteen were PIB- by blinded visual read. tpvCBS criteria had a sensitivity of 82% and a specificity of 71% for PIB+ status (Table 3) for all CBS patients, regardless of confidence ratings. In examining individual criteria within the variants, cognitive testing had the strongest association with PIB status, with OR of 8.1 for PIB+ in tpvCBS (episodic memory/visuospatial impairment greater than executive dysfunction) and OR of 6.4 for PIB- in fvCBS (executive dysfunction greater than memory/visuospatial impairment, *P* <0.05 for both). No other individual criterion was significantly associated with PIB results. There were no significant differences on individual neuropsychological measures when comparing CBS-PIB+ and CBS-PIB- at the group level. There was a trend (*P* = 0.08) for worse performance on the Benson figure copy in the CBS-PIB+ group and a trend (*P* = 0.07) for greater impairment in phonemic fluency in the CBS-PIB- group (see Additional file 2: Table S2). In patients who underwent ApoE genotyping, the presence of an ApoE4 allele was 90% specific but only 60% sensitive for PIB-positivity.

**Table 3:** **Comparison of methods versus PIB-PET results**

	**Criteria**	**FDG-Qual**	**FDG-Quant**	**MRI**
Number	25	23	22	24
True negative	10	6	7	6
True positive	9	10	9	8
False negative	2	1	1	3
False positive	4	6	5	7
Accuracy	0.76	0.70	0.73	0.58
Rater confidence rating	44%	74%	N/A	54%

Criteria = modified clinical criteria; True Negative = number of patients categorized as fvCBS who were PIB negative; True Positive = number of patients categorized as tpvCBS who were PIB positive; False Negative = number of patients categorized as fvCBS who were PIB positive; False Positive = number of patients categorized as tpvCBS who were PIB negative; Accuracy = True Negative + True Positive/total Number; Confidence Rating = number of highly confident/total number of patients; FDG-Qual, fluorodeoxyglucose PET qualitative (visual assessment); FDG-Quant, fluorodeoxyglucose PET quantitative (computed using ROI); MRI, magnetic resonance imaging; N/A, not applicable; PET, positron emission tomography; ROI, region of interest.

### Imaging

Structural MRI was available on all but one patient and FDG-PET was available on 23 (quantitative FDG-PET available on 22, one scan was excluded due to different PET scanner type and quantitative results were not directly comparable). The sensitivity of visual MRI reads for PIB+ status was 73% and specificity was 46%. Quantitative and qualitative readings of FDG-PET both yielded a sensitivity of 91% and similar specificities (58% and 50%, Table 3). Intra-rater reliability of FDG-PET qualitative reading was perfect (κ = 1.00) while MRI intra-rater reliability was modest (κ = 0.42). Confidence rating was highest for FDG-PET and lowest for clinical criteria, although these differences were not significant.

The combination of tpvCBS criteria and temporoparietal-predominant qualitative FDG-PET was a strong predictor for CBS-PIB+ (*P* = 0.005, OR 22.5 (95% confidence interval 2.6 to 194.5); see Additional file 3: Table S3). Combined tpvCBS criteria and temporoparietal-predominant MRI also predicted CBS-PIB+ (*P* <0.05, OR 9.6 (95% confidence interval 1.378 to 67.2); see Additional file 3: Table S3, which provides ORs for clinical criteria alone and in combination with FDG-PET and MRI for predicting PIB positivity and negativity).

**Figure 2:**
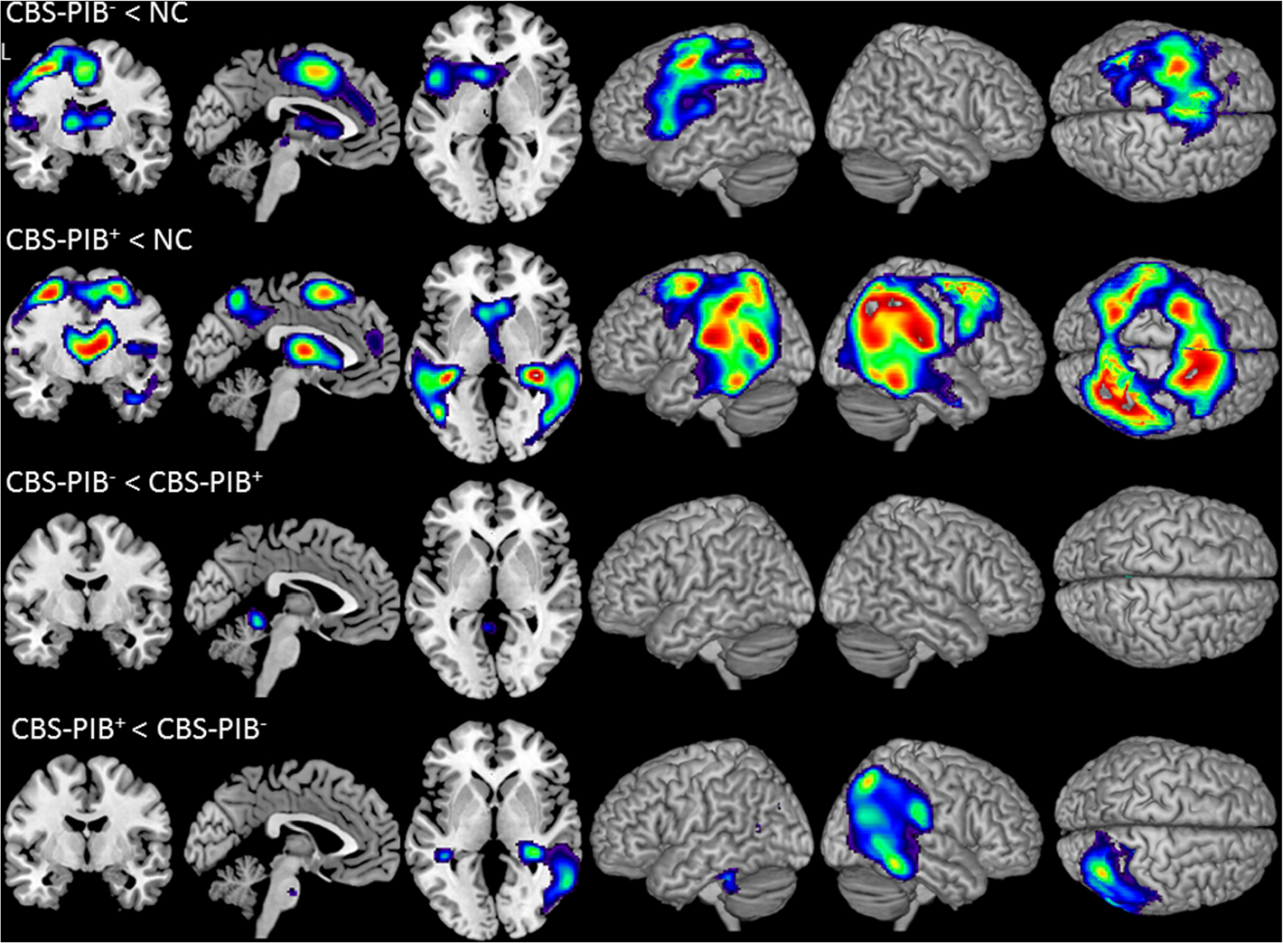
**Voxel-wise FDG comparisons.** Patterns of hypometabolism in CBS-PIB- and CBS-PIB+ compared with normal controls (NC) and compared with each other. Voxel-wise comparisons included sex, education and age as nuisance variables. T-score maps are rendered on the ch2.better template. All results are presented at a threshold of *P* <0.001, uncorrected for multiple comparisons. CBS-PIB-, corticobasal syndrome Pittsburgh compound negative; CBS-PIB+, corticobasal syndrome Pittsburgh compound B positive; FDG, fluorodeoxyglucose.

At a group level, the CBS-PIB+ and the CBS-PIB- cohorts both demonstrated hypometabolism compared with controls in peri-rolandic/posterior frontal regions on voxel-wise comparisons (left sided in CBS-PIB-, bilateral in CBS-PIB+, *P* <0.001 uncorrected, Figure 2). Decreased metabolism in both groups was noted in the caudate nucleus, although this signal should be interpreted with caution since these results may be, at least in part, due to ventricular enlargement in CBS patients versus controls. Hypometabolism in CBS-PIB+ extended into bilateral temporoparietal cortex.

On direct comparison of patient groups, hypometabolism was greater in the CBS-PIB- in a small cluster in the midline superior cerebellum, and greater in the CBS-PIB+ group in extensive regions of right temporoparietal cortex (Figure 2).

The CBS-PIB+ group had lower glucose metabolism than normal controls in the bilateral ‘common’ and temporoparietal and right frontal ROIs. The CBS-PIB- group had decreased uptake compared to controls in the bilateral ‘common’, left frontal and left temporoparietal ROIs (Table 4). CBS-PIB+ had greater hypometabolism than CBS-PIB- in the right temporoparietal ROI.

**Table 4:** **FDG ROI Z score and lateralization index comparisons**

	**CBS-PIB + (number = 10)**	**CBS-PIB-(number = 12)**	**NC (number = 26)**	***P***	***Post hoc***
FDG-common, z	−1.89 ± 0.81	−1.69 ± 1.37	0.00 ± 1.00	0.000	CBS-PIB+ versus NC *P* <0.001, CBS-PIB- versus NC *P* <0.001
right	−2.29 ± 0.89	−1.48 ± 1.66	0.00 ± 1.00	0.000	CBS-PIB+ versus NC *P* <0.001, CBS-PIB- versus NC *P* <0.01
left	−1.59 ± 0.91	−1.91 ± 1.30	0.00 ± 1.00	0.000	CBS-PIB+ versus NC *P* <0.001, CBS-PIB- versus NC *P* <0.001
LI	10.42 ± 4.37	8.82 ± 5.99	2.83 ± 1.72	0.000	CBS- PIB+ versus NC *P* <0.001, CBS-PIB- versus NC *P* <0.014
FDG-frontal, z	−1.26 ± 0.91	−1.06 ± 1.61	0.00 ± 1.00	0.005	CBS-PIB+ versus NC *P* <0.05, CBS-PIB- versus NC *P* <0.05
right	−1.51 ± 0.94	−0.83 ± 1.55	0.00 ± 1.00	0.002	CBS-PIB+ versus NC *P* <0.01
left	−1.00 ± 0.92	−1.30 ± 1.84	0.00 ± 1.00	0.008	CBS-PIB- versus NC *P* <0.05
LI	6.53 ± 3.08	10.52 ± 8.16	1.70 ± 1.33	0.000	CBS- PIB+ versus NC *P* <0.002, CBS-PIB- versus NC *P* <0.008
FDG-temporoparietal, z	−2.76 ± 1.65	−0.93 ± 1.65	0.00 ± 1.00	0.000	CBS-PIB+ versus CBS-PIB- *P* <0.01, CBS-PIB+ versus NC *P* <0.001
right	−3.22 ± 2.12	−0.65 ± 1.84	0.00 ± 1.00	0.000	CBS-PIB+ versus CBS-PIB- *P* <0.05, CBS-PIB+ versus NC *P* <0.05
left	−2.20 ± 1.38	−1.06 ± 1.56	0.00 ± 1.00	0.000	CBS-PIB+ versus NC *P* <0.001, CBS-PIB- versus NC *P* <0.05
LI	13.10 ± 8.20	8.45 ± 6.95	2.00 ± 1.41	0.000	CBS- PIB+ versus NC *P* <0.005, CBS-PIB- versus NC *P* <0.021

CBS-PIB+, corticobasal syndrome positive for Pittsburgh compound B; CBS-PIB-, corticobasal syndrome negative for Pittsburgh compound B; FDG, fluorodeoxyglucose; LI, lateralization index; NC, normal control; ROI, region of interest. Data are mean ± standard deviation.

FDG-PET LIs are shown in Table 4. CBS-PIB+ and the CBS-PIB- both showed greater lateralization of FDG uptake compared to controls in all ROIs, but there was no difference in lateralization between CBS-PIB+ and CBS-PIB-.

### Pathology

**Figure 3:**
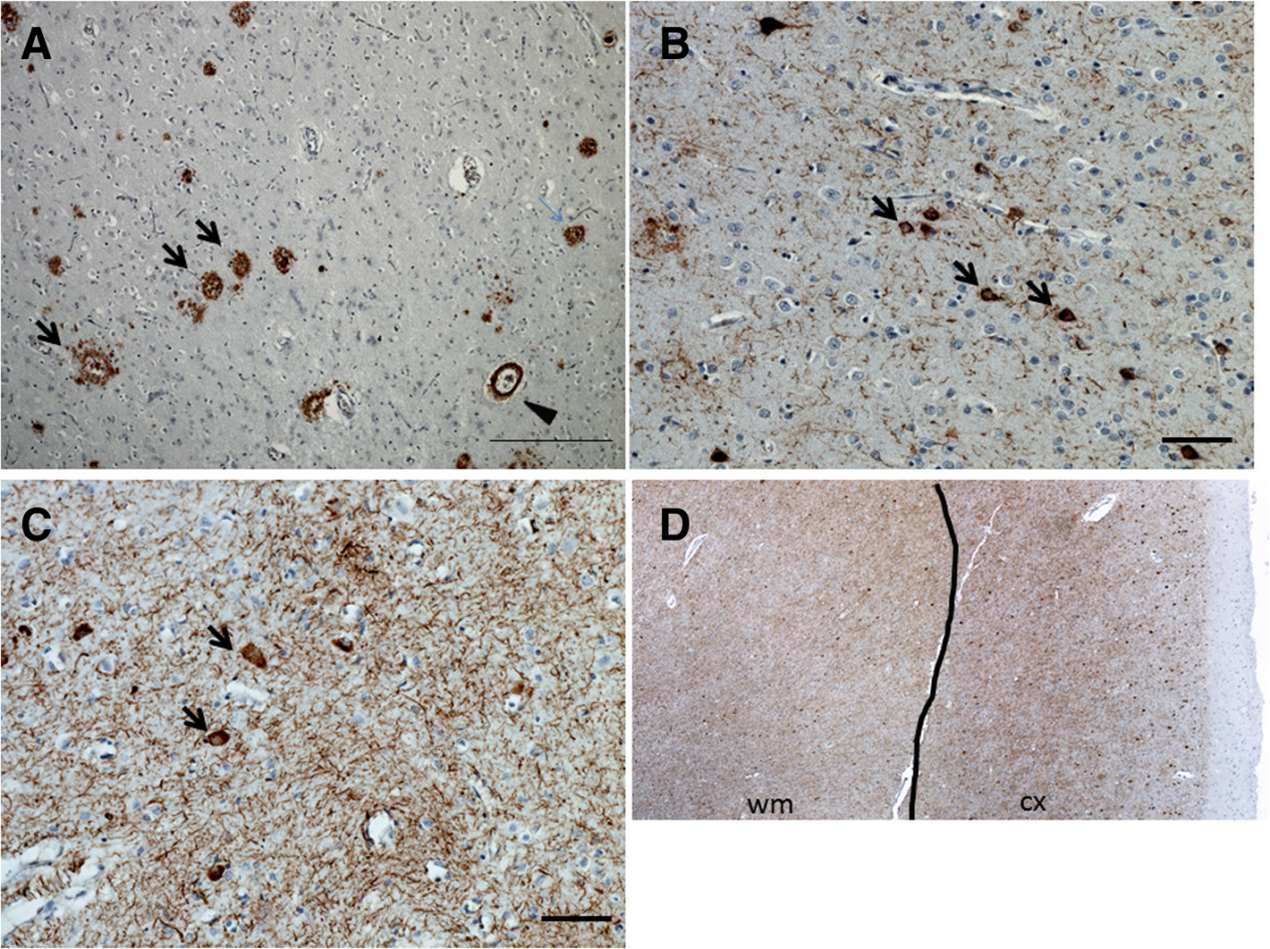
**Neuropathology of patient 13. A**. Angular gyrus immunostained against beta-amyloid (4G8). Arrows indicate neuritic plaques and arrowhead indicates cerebral amyloid angiopathy, a typical finding in Alzheimer’s disease patients (Scale bar: 250 μm); **B**. Inferior temporal gyrus immunostained against phospho-tau (CP-13). The arrows point to neurofibrillary tangles, characteristic of AD. Note their flame-like shape (Scale bar: 500 μm); **C**. Precentral gyrus immunostained against phospho-tau (CP-13). Arrows indicate ballooned neurons in the cortical deep layers, commonly seen in CBD (Scale bar: 500 μm); **D**. Precentral gyrus immunostained against phospho-tau (CP-13). The line divides the cortex to the right (cx) and the white matter to the left (WM). Note the striking positivity of white matter for phospho-tau, a feature seen in CBD but not in other tauopathies or AD. AD, Alzheimer’s disease; CBD, corticobasal degradation.

Autopsy (N = 7) or biopsy (N = 1) results were consistent with PIB-PET findings during life (Table 5). PIB-PET scans were positive in two patients who met criteria for both high probability AD (NIA-Reagan criteria) and intermediate probability Lewy body disease (McKeith criteria) [21,23]. A third CBS-PIB+ patient was found to have high probability AD (NIA-Reagan) and CBD at autopsy (with vascular brain injury as a contributing diagnosis). This patient had frequent neocortical neuritic plaques (CERAD score) and neurofibrillary tangles (Braak Stage VI) accompanied by tau-positive glial cytoplasmic inclusions (astrocytes and oligodendroglia) and subcortical white matter pathology consistent with CBD (Figure 3). CBD pathology was more restricted in topographical distribution than the widespread AD pathology, leading the pathologist to consider AD as the primary pathological diagnosis and CBD as a contributing secondary pathology. PIB-PET scans were negative in three patients with pathologically confirmed CBD, one with progressive supranuclear palsy (PSP) and one with both Pick’s disease and LBD.

**Table 5:** **Pathology results in comparison to other methods of assessment**

**Patient**	**PIB-PET**	**Pathology**	**Criteria**	**FDG-Qual**	**FDG-Quant**	**MRI**
2	-	CBD	fvCBS	tpvCBS	tpvCBS	fvCBS
3	-	CBD	fvCBS	fvCBS	fvCBS	fvCBS
8	-	CBD	tpvCBS	tpvCBS	tpvCBS	N/A
10	-	PSP	fvCBS	fvCBS	fvCBS	tpvCBS
14	-	Pick’s + DLB	tpvCBS	fvCBS	fvCBS	fvCBS
13	+	AD + CBD^a^	tpvCBS	tpvCBS	tpvCBS	tpvCBS
18	+	AD + DLB	tpvCBS	tpvCBS	tpvCBS	tpvCBS
19	+	AD + DLB	tpvCBS	tpvCBS	tpvCBS	fvCBS

^a^Patient 13 had evidence of AD and CBD pathology, but AD was the predominant pathology. AD, Alzheimer’s disease; CBD, corticobasal degeneration; fvCBS, frontal variant corticobasal syndrome; FDG-Qual, fluorodeoxyglucose PET qualitative (visual assessment); LBD, Lewy body disease; FDG-Quant, fluorodeoxyglucose PET quantitative (computed using ROI); PET, positron emission tomography; PSP, progressive supranuclear palsy; ROI, region of interest; tpvCBS, temporoparietal variant CBS. A ‘+’ or ‘-’ indicates positive or negative, respectively.

## Discussion

This study evaluated the utility of clinical criteria, FDG-PET and structural MRI in predicting the PIB-PET findings in 25 patients meeting core clinical criteria for CBS. Prior studies have assessed correlates of pathology in CBS at a group level, but our goal was to identify distinguishing predictors for individuals, particularly measures available to clinicians. FDG-PET (qualitatively and quantitatively) had the best sensitivity (approximately 90%) for predicting PIB+ status and proposed clinical criteria for tpvCBS had the best specificity (71%). Visual interpretation of structural MRI fared the worst and was not useful in determining PIB-status when used alone. Combining clinical tpvCBS criteria (specific) with FDG-PET (sensitive) yielded the best overall discrimination of PIB+ versus PIB- CBS patients.

Our proposed criteria offered diagnostic utility, yielding 76% classification accuracy. Sensitivity of tpvCBS criteria for CBS-PIB+ was high (82%), although specificity was modest (71%), suggesting that these criteria require further refinement. One main issue was difficulty in operationalization. Many patients met criteria for both variants, requiring clinical judgment for categorization. Not surprisingly, there was high confidence in fewer than half of patients. The individual criterion that was the best predictor of PIB status was the pattern of cognitive impairment. An executive-predominant pattern predicted PIB- and a memory/visuospatial predominant pattern predicted PIB+ status, mirroring results from group-level comparisons of autopsy-proven patients of CBS-FTLD versus CBS-AD [7,9]. In contrast to previous studies, non-fluent aphasia and behavioral presentations did not predict negative PIB. This may be due to the heterogeneous pathologies that likely underlie CBS-PIB-, including CBD, PSP and FTLD-TDP. Parietal features included in our tpvCBS criteria (for example, Gerstmann syndrome) also lacked predictive power, perhaps reflecting the proclivity of both AD and non-AD pathology to affect dorsolateral parietal cortex in CBS. In our cohort, anatomical overlap between PIB+ and PIB- patients was greater in the left parietal lobe than in right parietal or bilateral temporal cortex (Figure 2), consistent with the greater predictive power of visuospatial and episodic memory loss compared to dominant parietal features and reflected by the trend for worse performance on the Benson figure copy seen in the CBS-PIB+ cohort. Although asymmetry of clinical symptoms, imaging analyses and cognitive results are inherent in CBS, asymmetry is not predictive of underlying pathology, also noted in a prior study by our center [7].

An expert international group has recently proposed new diagnostic criteria for CBD [14]. The goal of these criteria differs from ours in a subtle, but important way: Armstrong and colleagues characterized clinical phenotypes predictive of CBD pathology, whereas we tested clinical features predictive of AD pathology in CBS patients. Unfortunately, the new criteria were published after completion of the chart review phase of our study, such that we were not able to test their reliability in identifying PIB-negative patients prospectively and in blinded fashion. Our core CBS criteria overlapped with Armstrong *et al*.’s for ‘probable CBS’ although we did not require asymmetry or apraxia as core diagnostic elements (however, lower limb apraxia which may have better anatomic specificity, was included in the frontal variant). The high prevalence of PIB+ patients in our cohort (44%) underscores the need to refine the definition of CBS in criteria that strive to be specific for underlying CBD. Notably, the Armstrong criteria recognize a ‘frontal behavioral-spatial’ syndrome and modified criteria from Cambridge also propose to lump together executive and visuospatial deficits as core cognitive features of CBS [32]. Our data suggest that the relative involvement of executive versus visuospatial dysfunction is a strong predictor of AD versus non-AD underlying neuropathology and these domains should be split in criteria that aim to predict underlying pathology in CBS.

Another important finding in our study was the ability to improve the prediction of PIB status for the individual by assessing whether the neurodegenerative pattern is predominantly frontal or temporoparietal on MRI or FDG-PET. FDG-PET showed high sensitivity for CBS-PIB+, perfect intra-rater agreement in visual interpretations, and high accuracy of visual reads versus quantitative analysis. These findings are consistent with previous studies demonstrating that FDG-PET is useful in differentiating AD from FTLD [18,33]. The combination of tpv-predominant clinical criteria and FDG-PET was particularly powerful, predicting PIB+ status with an OR of 22.5. In lieu of access to amyloid imaging or other biomarkers, FDG-PET may be a useful ancillary test for differentiating pathologies in CBS. However, a temporoparietal pattern on FDG was not specific for PIB+ scans. While frontal-predominant hypometabolism is uncommon in CBS patients with AD, a temporoparietal-predominant pattern does not exclude the possibility of non-AD pathology. Visual interpretation of MRI atrophy patterns proved less helpful. Quantitative MRI measures would likely have performed better, but were not included in this study because patients underwent MRI on 1.5 T, 3 T and 4 T scanners. Our study suggests that structural and functional imaging markers can improve diagnostic accuracy when supplementing clinical criteria and may prove useful in future iterations of criteria.

Although the aim of this study was to determine predictors of PIB-PET status for the individual, we also performed voxel-wise and specific ROI group analyses. Our findings comparing CBS-PIB+ with CBS-PIB- were similar to prior reports contrasting autopsy-confirmed patients of CBS-AD versus CBS-FTLD [7,10,11,34,35]. CBS-PIB+ had greater temporoparietal hypometabolism than CBS-PIB-, and both groups showed hypometabolism in posteromedial frontal and peri-rolandic cortex, regions that comprise a sensorimotor integration network [36]. This network may be the core affected network in all patients with the CBS phenotype, independent of underlying pathology. The contrast CBS-PIB- < CBS-PIB+ did not yield a characteristic FDG pattern, likely due to the heterogeneous pathologies represented in this group. Interestingly, we found greater hypometabolism in CBS-PIB- in the medial cerebellum, a region often affected in PSP, which may be the second most common cause of CBS [7]. Asymmetry did not distinguish CBS-PIB+ from CBS-PIB- and does not aid in predicting pathology, consistent with our observations in pathologically-confirmed CBS-AD and CBS-FTLD [7]. However, as a group, CBS-PIB+ had a right-sided bias for metabolic patterns and CBS-PIB- had a left sided bias which may reflect the inclusion of non-fluent variant primary progressive aphasia (PPA) patients in the CBS-PIB- cohort.

Our study, as well as another study applying amyloid PET in CBS [13], found similar clinical and anatomical differences between CBS-PIB+ and CBS-PIB- to those reported when comparing CBS-AD and CBS-FTLD in clinicopathological studies, providing preliminary validation for the use of amyloid imaging as an *in vivo* proxy for AD pathology in CBS. However, as illustrated by one of our autopsy-proven patients, a positive amyloid PET scan does not exclude the presence of CBD or another meaningful co-pathology, although in this case CBD was considered secondary to AD in driving the clinical syndrome of CBS. In fact, ratings were tpvCBS on all methods of assessment suggesting underlying AD pathology. Importantly, significant neuritic plaques (and positive amyloid PET) can also be seen in 15% to 30% of cognitively normal older individuals [37]. Therefore, while a negative amyloid scan ‘rules out’ AD with high likelihood in patients presenting with CBS, a positive scan does not exclude a co-pathology that may be the primary etiology of symptoms, particularly in patients with frontal features which are most strongly associated with underlying FTLD pathology.

Aside the from lack of pathological confirmation in some patients, limitations of our study include the relatively small sample size (although our cohort was similar in size to previous studies of CBS which is an uncommon syndrome) and the retrospective chart and imaging review by a single rater. It is possible that there was a referral bias to our cognitive clinic, rather than a movement disorder one. However, our clinic and research group receives primary movement disorders patient referrals, particularly to our cohort research studies. Additionally, this study was designed to build upon our prior study [7] that found the nature of severity of the movement disorder did not distinguish AD from FTLD, stressing the importance of a multi-disciplinary approach to the evaluation of CBS. The criteria for each variant were based on our review of the literature and were not intended to provide final or validated criteria. Rather, this study was designed to examine whether splitting CBS into clinical variants could improve our ability to predict PIB status. A similar approach has been fruitful in PPA [16]. The criteria presented here can, therefore, be considered a foundation upon which to build future consensus criteria. While the future evaluation of CBS may incorporate molecular biomarkers for amyloid beta (Aβ), tau [38] and TDP-43 [39], even the most validated of these markers (cerebrospinal fluid Aβ and tau and amyloid PET) are not yet accessible in routine clinical practice despite a decade or more of research. Tau PET scans are the subject of current research [38] and will likely be incorporated in the future with amyloid PET to predict pathology for the individual patient. Possible implications include inclusion/exclusion criteria for clinical trials, ruling out co-pathology, or biomarker progression of disease. Determining the presence of co-pathology with amyloid potentially could alter treatment efficacy or disease course of a patient and would be important to note when analyzing results of a study or trial. For the moment, access to these markers is largely confined to the research realm, where they can be applied to refine clinical criteria and optimize the integration of more accessible tools, such as MRI and FDG-PET, into the diagnostic assessment of CBS and other heterogeneous disorders [40].

## Conclusions

This study was not designed to identify cost-effectiveness, but it examined approaches to optimize suspected underlying pathologies to support the potential of specific targeted treatment. Our data suggest that there is current diagnostic value in ‘splitting’ CBS patients into those presenting with predominant frontal features (who are likely to have underlying FTLD pathology) and those with predominant temporoparietal features (which are highly sensitive although not specific for underlying AD). The predictive value can be enhanced by classifying the predominant pattern of neurodegeneration on neuroimaging. In practice, MRI is the standard of care and will likely be acquired in most patients. However, in our studies atrophy patterns on MRI were often hard to classify with confidence as ‘frontal’ or ‘temporoparietal’ predominant. In such uncertain cases, the addition of FDG-PET can be diagnostically useful. As molecular imaging is increasingly validated and accessible, future diagnostic algorithms in CBS will likely include a combination of amyloid and tau PET in addition to clinical assessment and MRI. This combination should allow the *in vivo* classification of CBS due to AD, primary tauopathy and (by process of elimination) FTLD-TDP pathology with high certainty, paving the way for the development and ultimate implementation of protein-specific therapies.

## Additional files

Additional file 1: Table S1. Diagnostic Criteria. This table demonstrates which diagnostic criterion is met for each patient in the CBS cohort, final diagnostic variant designation, confidence rating, and PIB-PET status. Additional file 2: Table S2. Neuropsychological results at first evaluation. This table compares the CBS-PIB- and CBS-PIB+ neuropsychological test results for each cognitive test and their respective *P* values. Additional file 3: Table S3. Combination of factors that best predict PIB positivity. This table provides odds ratios for clinical criteria alone and in combination with FDG-PET and MRI for predicting PIB positivity.

## Abbreviations

AAL: Automated anatomical labeling
AD: Alzheimer’s disease
CBD: Corticobasal degeneration
CBS: Corticobasal syndrome
CBS-AD: Corticobasal syndrome due to Alzheimer’s disease
CBS-FTLD: Corticobasal syndrome due to frontotemporal lobar dementia pathology
CBS-PIB-: Corticobasal syndrome Pittsburgh compound negative
CBS-PIB+: Corticobasal syndrome Pittsburgh compound B positive
DVR: Distribution volume ratios
FDG-PET: Fluorodeoxyglucose positron emission tomography
FTLD-TDP: Frontotemporal lobar degeneration with TDP-43 inclusions
Fv: Frontal variant
fvCBS: Frontal variant corticobasal syndrome
LBD: Lewy body disease
LBNL: Lawrence Berkeley National Laboratory
LI: Lateralization indices
MMSE: Mini Mental State Exam
NC: Normal controls
OR: Odds ratio
PIB-PET: Pittsburgh compound B positron emission tomography
PPA: Primary progressive aphasia
ROI: Regions of interest
SUVR: Standard uptake volume ratios
Tpv: Temporoparietal variant
tpvCBS: Temporoparietal-predominant variant corticobasal syndrome
UCSF: University of California San Francisco

## References

[1] Gibb WR, Luthert PJ, Marsden CD (1989). Corticobasal degeneration. Brain.

[2] Riley DE, Lang AE, Lewis A, Resch L, Ashby P, Hornykiewicz O (1990). Cortical-basal ganglionic degeneration. Neurology.

[3] Rebeiz JJ, Kolodny EH, Richardson EP (1968). Corticodentatonigral degeneration with neuronal achromasia. Arch Neurol.

[4] Chand P, Grafman J, Dickson D, Ishizawa K, Litvan I (2006). Alzheimer’s disease presenting as corticobasal syndrome. Mov Disord.

[5] Doran M, Du Plessis D, Enevoldson T, Fletcher N, Ghadiali E, Larner A (2003). Pathological heterogeneity of clinically diagnosed corticobasal degeneration. J Neurol Sci.

[6] Alladi S, Xuereb J, Bak T, Nestor P, Knibb J, Patterson K (2007). Focal cortical presentations of Alzheimer’s disease. Brain.

[7] Lee SE, Rabinovici GD, Mayo MC, Wilson SM, Seeley WW, Dearmond SJ (2011). Clinicopathological correlations in corticobasal degeneration. Ann Neurol.

[8] Boeve BF, Maraganore DM, Parisi JE, Ahlskog JE, Graff-Radford N, Caselli RJ (1999). Pathologic heterogeneity in clinically diagnosed corticobasal degeneration. Neurology.

[9] Shelley BP, Hodges JR, Kipps CM, Xuereb JH, Bak TH (2009). Is the pathology of corticobasal syndrome predictable in life?. Mov Disord.

[10] Josephs KA, Whitwell JL, Boeve BF, Knopman DS, Petersen RC, Hu WT (2010). Anatomical differences between CBS-corticobasal degeneration and CBS-Alzheimer’s disease. Mov Disord.

[11] Whitwell J, Jack C, Boeve B, Parisi J, Ahlskog J, Drubach D (2010). Imaging correlates of pathology in corticobasal syndrome. Neurology.

[12] Hu WT, Rippon GW, Boeve BF, Knopman DS, Petersen RC, Parisi JE (2009). Alzheimer’s disease and corticobasal degeneration presenting as corticobasal syndrome. Mov Disord.

[13] Burrell JR, Hornberger M, Villemagne VL, Rowe CC, Hodges JR (2013). Clinical profile of PiB-positive corticobasal syndrome. PLoS One.

[14] Armstrong MJ, Litvan I, Lang AE, Bak TH, Bhatia KP, Borroni B (2013). Criteria for the diagnosis of corticobasal degeneration. Neurology.

[15] Klunk WE, Engler H, Nordberg A, Wang Y, Blomqvist G, Holt DP (2004). Imaging brain amyloid in Alzheimer’s disease with Pittsburgh Compound-B. Ann Neurol.

[16] Gorno-Tempini ML, Hillis AE, Weintraub S, Kertesz A, Mendez M, Cappa SF (2011). Classification of primary progressive aphasia and its variants. Neurology.

[17] Mormino E, Kluth J, Madison C, Rabinovici G, Baker S, Miller B (2009). Episodic memory loss is related to hippocampal-mediated β-amyloid deposition in elderly subjects. Brain.

[18] Rabinovici G, Rosen H, Alkalay A, Kornak J, Furst A, Agarwal N (2011). Amyloid vs FDG-PET in the differential diagnosis of AD and FTLD. Neurology.

[19] Boxer AL, Geschwind MD, Belfor N, Gorno-Tempini ML, Schauer GF, Miller BL (2006). Patterns of brain atrophy that differentiate corticobasal degeneration syndrome from progressive supranuclear palsy. Arch Neurol.

[20] Ellison D, Love S, Chimelli LM, Harding B, Lowe JS, Vinters HV (2013). Neuropathology: a reference text of CNS pathology.

[21] Consensus recommendations for the postmortem diagnosis of Alzheimer’s disease. The National Institute on Aging, and Reagan Institute Working Group on Diagnostic Criteria for the Neuropathological Assessment of Alzheimer’s Disease. Neurobiol Aging. 1997;18:S1-2. 9330978

[22] Dickson DW, Bergeron C, Chin SS, Duyckaerts C, Horoupian D, Ikeda K (2002). Office of Rare Diseases neuropathologic criteria for corticobasal degeneration. J Neuropathol Exp Neurol.

[23] McKeith IG, Dickson DW, Lowe J, Emre M, O’Brien JT, Feldman H (2005). Diagnosis and management of dementia with Lewy bodies: third report of the DLB Consortium. Neurology.

[24] Bettcher BM, Wilheim R, Rigby T, Green R, Miller JW, Racine CA (2012). C-reactive protein is related to memory and medial temporal brain volume in older adults. Brain Behav Immun.

[25] Rosen HJ, Gorno-Tempini ML, Goldman WP, Perry RJ, Schuff N, Weiner M (2002). Patterns of brain atrophy in frontotemporal dementia and semantic dementia. Neurology.

[26] Zhang Y, Schuff N, Ching C, Tosun D, Zhan W, Nezamzadeh M (2011). Joint assessment of structural, perfusion, and diffusion MRI in Alzheimer’s disease and frontotemporal dementia. Int J Alzheimers Dis.

[27] SPM. http://www.fil.ion.ucl.ac.uk/spm.

[28] Logan J, Fowler JS, Volkow ND, Wang GJ, Ding YS, Alexoff DL (1996). Distribution volume ratios without blood sampling from graphical analysis of PET data. J Cereb Blood Flow Metab.

[29] Price JC, Klunk WE, Lopresti BJ, Lu X, Hoge JA, Ziolko SK (2005). Kinetic modeling of amyloid binding in humans using PET imaging and Pittsburgh Compound-B. J Cereb Blood Flow Metab.

[30] Lehmann M, Ghosh PM, Madison C, Laforce R, Corbetta-Rastelli C, Weiner MW (2013). Diverging patterns of amyloid deposition and hypometabolism in clinical variants of probable Alzheimer’s disease. Brain.

[31] Tzourio-Mazoyer N, Landeau B, Papathanassiou D, Crivello F, Etard O, Delcroix N (2002). Automated anatomical labeling of activations in SPM using a macroscopic anatomical parcellation of the MNI MRI single-subject brain. Neuroimage.

[32] Mathew R, Bak TH, Hodges JR (2012). Diagnostic criteria for corticobasal syndrome: a comparative study. J Neurol Neurosurg Psychiatry.

[33] Foster NL, Heidebrink JL, Clark CM, Jagust WJ, Arnold SE, Barbas NR (2007). FDG-PET improves accuracy in distinguishing frontotemporal dementia and Alzheimer’s disease. Brain.

[34] Ukmar M, Moretti R, Torre P, Antonello R, Longo R, Bava A (2003). Corticobasal degeneration: structural and functional MRI and single-photon emission computed tomography. Neuroradiology.

[35] Koyama M, Yagishita A, Nakata Y, Hayashi M, Bandoh M, Mizutani T (2007). Imaging of corticobasal degeneration syndrome. Neuroradiology.

[36] Seeley WW, Crawford RK, Zhou J, Miller BL, Greicius MD (2009). Neurodegenerative diseases target large-scale human brain networks. Neuron.

[37] Laforce R, Rabinovici GD (2011). Amyloid imaging in the differential diagnosis of dementia: review and potential clinical applications. Alzheimers Res Ther.

[38] Maruyama M, Shimada H, Suhara T, Shinotoh H, Ji B, Maeda J (2013). Imaging of tau pathology in a tauopathy mouse model and in Alzheimer patients compared to normal controls. Neuron.

[39] Geser F, Prvulovic D, O’Dwyer L, Hardiman O, Bede P, Bokde A (2011). On the development of markers for pathological TDP-43 in amyotrophic lateral sclerosis with and without dementia. Prog Neurobiol.

[40] McKhann GM, Knopman DS, Chertkow H, Hyman BT, Jack CR, Kawas CH (2011). The diagnosis of dementia due to Alzheimer’s disease: recommendations from the National Institute on Aging-Alzheimer’s Association workgroups on diagnostic guidelines for Alzheimer’s disease. Alzheimers Dement.

