# Supplementary material for: Predicting amyloid status in corticobasal syndrome using modified clinical criteria, magnetic resonance imaging and fluorodeoxyglucose positron emission tomography
Source: Alzheimers Res Ther. 2015 Mar 2;7(1):8. doi: 10.1186/s13195-014-0093-y (PMC4346122; doi:10.1186/s13195-014-0093-y)
Supplement: Additional file 1: Table S1. — Diagnostic Criteria. This table demonstrates which diagnostic criterion is met for each patient in the CBS cohort, final diagnostic variant designation, confidence rating, and PIB-PET status. [file 13195_2014_93_MOESM1_ESM.docx]

Supplemental Table e1. Diagnostic Criteria

| Patient | fv-NF | fv-Apathy | fv-Apraxia | fv-Ex | pv-LPA | pv-Gerst | pv-Memory | Criteria | Confidence | PIB-PET |
| --- | --- | --- | --- | --- | --- | --- | --- | --- | --- | --- |
| 1 | - | + | - | + | - | + | - | fvCBS | 3 | - |
| 2 | - | - | - | + | + | - | - | fvCBS | 3 | - |
| 3 | - | + | - | + | + | + | - | fvCBS | 3 | - |
| 4 | - | + | - | + | - | - | - | fvCBS | 3 | - |
| 5 | - | - | - | - | - | - | + | tpvCBS | 3 | - |
| 7 | - | - |  | + | - |  | - | fvCBS | 2 | - |
| 8 | - | - | - | - | + | + | + | tpvCBS | 4.5 | - |
| 9 | + | - |  | + | - | - | - | fvCBS | 2 | - |
| 10 | + | + |  | + | - | - | - | fvCBS | 1 | - |
| 12 | + | - |  | + | - | - | - | fvCBS | 2.5 | - |
| 14 | - | - |  | + | + | + | + | tpvCBS | 4 | - |
| 15 | + | + | - | + | - | - | - | fvCBS | 1.5 | - |
| 17 | - | + |  | + | - | + | + | tpvCBS | 3.5 | - |
| 25 | + | + |  | - | - | - | + | fvCBS | 2 | - |
| 6 | + | - | + | - | - | - | + | tpvCBS | 3 | + |
| 11 | - | + | - | - | - | - | + | fvCBS | 3 | + |
| 13 | - | - |  | - | - | + | + | tpvCBS | 5 | + |
| 16 | + | + | + | + | + | + | + | tpvCBS | 3.5 | + |
| 18 | - | - |  | - | - | + | + | tpvCBS | 4.5 | + |
| 19 | - | + |  | + | - | - | - | tpvCBS | 3.5 | + |
| 20 | - | - |  | - | - | + | + | tpvCBS | 4 | + |
| 21 | + | + | + | - | - | - | + | fvCBS | 3 | + |
| 22 | - | - |  | + | - | + | - | tpvCBS | 3 | + |
| 23 | - | - |  | - | - | + | + | tpvCBS | 5 | + |
| 24 | + | + | + | + | + | - | + | tpvCBS | 3.5 | + |

Legend:

fv-NF=CBS frontal variant “Agrammatism, non-fluent, or motor speech deficits”; fv-Apathy= CBS frontal variant “Apathy, disinhibition, or loss of empathy”; fv-Apraxia= CBS frontal variant “Apraxia primarily affecting lower extremities”; fv-Exec= CBS frontal variant “Prominent executive dysfunction greater than memory or visuospatial impairment”; Criteria= diagnostic assignment by criteria alone; tpv-LPA= CBS temporoparietal variant “Logopenic aphasia (2)”; tpv-Gerst= CBS temporoparietal variant “Elements of Gerstmann or Balint syndrome”; tpv-Mem= CBS temporoparietal variant “Episodic memory or visuospatial impairment greater than executive dysfunction”. A ‘+’ or ‘-’ indicates presence or absence of category, respectively. Insufficient data was left blank.

References:

(1) Gorno-Tempini ML, Hillis AE, Weintraub S, Kertesz A, Mendez M, Cappa SF, et al. Classification of primary progressive aphasia and its variants. Neurology 2011 Mar 15;76(11):1006-1014.
