# Supplementary material for: Predicting amyloid status in corticobasal syndrome using modified clinical criteria, magnetic resonance imaging and fluorodeoxyglucose positron emission tomography
Source: Alzheimers Res Ther. 2015 Mar 2;7(1):8. doi: 10.1186/s13195-014-0093-y (PMC4346122; doi:10.1186/s13195-014-0093-y)
Supplement: Additional file 2: Table S2. — Neuropsychological results at first evaluation. This table compares the CBS-PIB- and CBS-PIB+ neuropsychological test results for each cognitive test and their respective P values. [file 13195_2014_93_MOESM2_ESM.docx]

Supplemental Table e2. Neuropsychological results at first evaluation

| **Test** | **CBS-PIB-** | **CBS-PIB+** | ***p*** |
| --- | --- | --- | --- |
| MMSE total | 25.0 (3.3) | 23.6 (6.7) | 0.796 |
| Benson figure copy | 12.8 (2.8) | 6.7 (6.9) | 0.080* |
| Benson figure delayed recall | 7.1 (4.5) | 4.9 (5.2) | 0.283 |
| Calculations | 3.4 (1.6) | 3.1 (1.9) | 0.781 |
| Modified Trails, lines correct | 11.3 (4.5) | 9.0 (5.5) | 0.315 |
| Modified Trails, errors | 2.1 (2.0) | 1.1 (1.5) | 0.312 |
| Phonemic fluency | 5.9 (4.1) | 9.0 (4.0) | 0.072* |
| Category fluency | 10.5 (5.6) | 11.7 (5.1) | 0.569 |
| Stroop interference correct | 21.8 (12.5) | 21.4 (14.5) | 0.952 |
| Stroop interference errors | 1.8 (4.8) | 3.0 (3.3) | 0.328 |
| CVLT total over 4 trials | 22.0 (6.9) | 18.9 (8.0) | 0.346 |
| CVLT delayed recall | 5.0 (2.9) | 3.9 (3.3) | 0.411 |
| CLVT recognition | 7.5 (2.0) | 7.8 (2.1) | 0.764 |
| Boston Naming Test | 11.5 (3.8) | 11.7 (3.9) | 0.865 |
| GDS total | 10.5 (7.8) | 7.1 (2.7) | 0.376 |

Legend:

Means reported with standard deviation in parentheses. *Trends reported at *p*<0.1. CVLT=California Verbal Learning Test; GDS=Geriatric Depression Scale; MMSE=Mini-Mental State Exam; NS=non-significant
