# Supplementary material for: Predicting amyloid status in corticobasal syndrome using modified clinical criteria, magnetic resonance imaging and fluorodeoxyglucose positron emission tomography
Source: Alzheimers Res Ther. 2015 Mar 2;7(1):8. doi: 10.1186/s13195-014-0093-y (PMC4346122; doi:10.1186/s13195-014-0093-y)
Supplement: Additional file 3: Table S3. — Combination of factors that best predict PIB positivity. This table provides odds ratios for clinical criteria alone and in combination with FDG-PET and MRI for predicting PIB positivity. [file 13195_2014_93_MOESM3_ESM.docx]

Supplemental Table e3. Combination of factors that best predict PIB positivity

| **Clinical criteria** | **FDG-Qual** | **MRI** | **Odds ratio** | **95% Confidence interval** | **P value** |
| --- | --- | --- | --- | --- | --- |
| **Meets criteria for tpvCBS** | **FDG-qual= tpv** | **MRI=tpv** |  |  |  |
| + |  |  | 11.3 | 1.65-76.9 | <0.05 |
| + | + |  | 20.0 | 2.29-175.0 | <0.01 |
| + |  | + | 9.6 | 1.37-67.2 | <0.05 |
| + | + | + | 17.5 | 1.60-191.9 | <0.05 |
| **Meets criteria for fvCBS** | **FDG-qual= fv** | **MRI-fv** |  |  |  |
| + |  |  | 0.1 | 0.02-0.81 | <0.05 |
| + | + |  | 0.2 | 0.008-4.3 | N.S. |
| + |  | + | 0.4 | 0.03-4.8 | N.S. |
| + | + | + | 0.3 | 0.01-8.3 | N.S. |

Legend:

Criteria= final designation of tpvCBS or fvCBS given based upon our modified clinical CBS criteria; FDG-Qual= fluorodeoxyglucose PET qualitative (visual assessment)
